# COVID-19 and Cardiac Implications—Still a Mystery in Clinical Practice

**DOI:** 10.31083/j.rcm2405125

**Published:** 2023-04-24

**Authors:** Reka Borka Balas, Lorena Elena Meliț, Cristina Oana Mărginean

**Affiliations:** ^1^Department of Pediatrics I, “George Emil Palade'' University of Medicine, Pharmacy, Sciences and Technology, 540136 Târgu Mureș, Romania

**Keywords:** children, COVID-19, cardiac injury, myocarditis

## Abstract

Although initially the evolution of Coronavirus disease 2019 (COVID-19) seemed less severe in pediatric patients, in the three years since the beginning of the pandemics, several severe cases have been described, pediatric inflammatory multisystem syndrome (PIMS) has been defined, pathogenesis is being continuously studied, and many aspects regarding the long-term evolution and multi-organ damage are still unexplained. Cardiac injuries in COVID-19 represent most-likely the second cause of mortality associated with the infection. A wide-spectrum of cardiac abnormalities were reported to be associated with COVID-19 in children including ventricular dysfunction, acute myocardial dysfunction, arrhythmias, conduction abnormalities, coronary artery dilation or aneurysms, and less common pericarditis and valvulitis. Risk factors for severe COVID-19 in children should be identified, laboratory tests and imaging techniques should be performed to reveal cardiac injury as soon as possible. The aim of this review was to highlight the great value of repeated cardiological monitoring in patients with COVID-19, underlining also the peculiarities in terms of pediatric population. This review is looking for answers on questions like ‘Why do some, but not all, patients with COVID-19 develop cardiac injury or severe hyperinflammatory status?’, ‘Which factors are involved in triggering COVID-19 associated cardiac injury?’, ‘What are the mechanisms involved in the etiology of cardiac injury?’, ‘Is there a clear relationship between hyperinflammation and cardiac injury?’, ‘Is hyperinflammatory status the pre-stage of cardiac injury in COVID-19 patients?’ which still lack clear answers. The understanding of mechanisms involved in the development of COVID-19 associated cardiac injury might shed light on all the above-mentioned mysteries and might increase the likelihood of favorable evolution even in severe cases.

## 1. Introduction

Coronavirus disease 2019 (COVID-19) is a relatively new disorder caused by severe acute respiratory syndrome coronavirus 2 (SARS-CoV-2) being no longer a novelty that is has been discovered in the late 2019 in Wuhan, China where it initially caused a cluster of severe cases of pneumonia associated with acute respiratory distress syndrome and it has spreads worldwide rapidly forcing the World Health Organization to declare a pandemic on 11th of March 2020 [1]. As of this writing, more than 600 million of people were infected with SARS-CoV-2, and the disease caused over 6.5 million deaths worldwide [2]. Although initially pediatric patients were thought to be spared of this infection or that they would develop only mild forms of infection, time proved the contrary highlighting that severe forms of COVID-19 might also happen in small patients. The first reports of severely affected children by COVID-19 emerged from the United Kingdom revealing several cases with symptoms resembling Kawasaki disease, macrophage activation syndrome, toxic shock syndrome or bacterial sepsis suggesting a severe hyperinflammatory state associated with SARS-CoV-2 infection [3]. Since this original reports, similar reports increased worldwide, including Romania [4, 5]. This syndrome was referred as multisystem inflammatory syndrome in children (MIS-C), pediatric multisystem inflammatory syndrome or pediatric inflammatory multisystem syndrome [6, 7, 8] and among SARS-CoV-2 infection forms it is most-likely the one with the greatest impact on cardiovascular system [1]. Most of these reports included previously healthy children, with asthma and obesity as frequently encountered comorbidities [4, 6, 9].

Aside from this severe syndrome, COVID-19 gained importance in cardiology for several reasons such as its potential to affects and impact the cardiovascular system; its related morbidity and mortality rates in patients with acquires cardiopathies; its associated symptoms that might resemble to symptoms triggered by cardiovascular diseases including shortness of breath or cyanosis, commonly encountered in forms of worsening cardiac disease; its well-known negative impact on patients with congenital heart diseases; and least, but not last the associated negative impact on the financial support offered to patients with cardiovascular disease [10]. Unsurprisingly, the involvement of a cardiology team in the management and follow-up of patients with SARS-CoV-2 infection regardless of the patient’s age is crucial for achieving the best outcome in clinical practice. Although, respiratory failure remains the main cause of death in patients with COVID-19, it is important to acknowledge that cardiac dysfunction has been stated as the second most important cause of mortality and it definitely requires major attention during admission [11, 12]. Therefore, the assessment of cardiac markers such as troponin I along with electro-cardiac changes might change the patient’s course especially in severe cases since they were noticed to occur in 7–17% of the hospitalized patients being predictable for acute cardiac injury [12].

Although almost three years have passed since the beginning of COVID-19 pandemic era, multiple shadows of unknowledge darken the prognosis of these patients and each report published on this topic might add valuable information in clinical practice. Thus, questions like ‘Why do some, but not all, patients with COVID-19 develop cardiac injury or severe hyperinflammatory status?’, ‘Which factors are involved in triggering COVID-19 associated cardiac injury?’, ‘What are the mechanisms involved in the etiology of cardiac injury?’, ‘Is there a clear relationship between hyperinflammation and cardiac injury?’, ‘Is hyperinflammatory status the pre-stage of cardiac injury in COVID-19 patients?’ still lack clear answers and will definitely benefit from more research in this area. Nevertheless, it is clear that all patients with COVID-19 regardless of the age or comorbidity should be screened for acute cardiac injury especially if hospitalized in order to preempt the irreversible damage or the associated life-threatening risks. The understanding of mechanisms involved in the development of COVID-19 associated cardiac injury might shed light on all the above mentioned mysteries and might increase the likelihood of favorable evolution even in severe cases.

Several mechanisms seem to be involved in COVID-19 associated cardiac injury such as (1) cardiomyocyte injury triggered by a severe acute inflammatory response evolving during well-documented COVID-19 induced cytokine storm, (2) cardiomyocytes cellular damage due to viral invasion, and (3) ischemic injury associated to severe hypoxia resulting from acute lung injury [10]. A wide-spectrum of cardiac abnormalities were reported to be associated with COVID-19 including ventricular dysfunction/acute myocardial dysfunction, arrhythmias, conduction abnormalities, coronary artery dilation or aneurysms, and less common pericarditis and valvulitis [6]. In fact, most of these findings might also be found in Kawasaki disease patients. Regardless of the variety of cardiac findings associated or triggered by SARS-CoV-2 infection, we must mention that most of the patients, especially children without previous comorbidities recover completely if properly managed. Therefore, the aim of this review was to highlight the great value of repeated cardiological monitoring in patients with COVID-19, underlining also the peculiarities in terms of pediatric population.

## 2. A Holistic Approach of the Relationship between SARS-CoV-2 and Cardiac Injuries

It is well-known at this point that SARS-CoV-2 virus enters the cells due to its ability of binding to the angiotensin converting enzyme 2 (ACE-2) through endocytosis [13, 14, 15, 16]. Taking into account that the ACE-2 is expressed on the epithelial surface of both alveoli and small intestine explaining the respiratory and gastrointestinal symptoms triggered by this infection [16, 17]. Even more important is this enzyme is also expressed in renal and cardiovascular tissues, as well as vascular endothelium [18]. Moreover, angiotensin 2 is known for its ability to promote lung injuries, but this effect is controlled by ACE-2, which in fact has the role to deactivate angiotensin 2 [19]. Unfortunately, in the setting of SARS-CoV-2, ACE-2 is no longer available due to viral binding and thus, angiotensin 2 will exert its role in the development of severe lung injury associated with COVID-19 [15, 17].

### 2.1 Pathogenesis

Cardiotoxicity represents the primary mechanism involved in the development of COVID-19 associated cardiac injuries and it is a multifactorial process involving the entry of the virus into cardiac cells due to the presence of ACE-2 on their surface as we already mentioned, COVID-19 associated hypoxia, and the side effects of the drugs used for this infection [20]. The direct viral invasion was sustained by previous studies which noticed the presence of viral RNA in 35% of the patients infected with other coronaviruses who died from myocardial infarction [20, 21, 22]. Based on the well-known children’s peculiarities related to the ACE-2 receptor distribution and the cellular viral invasion, we might explain the children’s lower susceptibility of developing severe forms of COVID-19 when compared to adults [23, 24].

The second most important mechanism is represented by the immune-mediated reactions secondary to the overproduction of cytokines or dysregulation of T cells triggered by the virus itself, both leading to endothelial dysfunction and microvascular damage [21, 25, 26, 27]. Moreover, the studies performed on animals pointed out that the increased level of cytokines along with the overproduction of inflammatory mediators might be involved in decreasing cardiac contractility via calcium-dependent pathways [28, 29, 30]. Another important role of the cytokine storm is related to the maintenance of hemodynamic instability due to their ability to cause peripheral vasodilation [28, 31, 32, 33, 34, 35]. In fact, this immune-mediated mechanism is the core of MIS-C and this hypothesis is sustained in several reports that mention complete recovery of the cardiac injuries after therapy with immunomodulatory agents such as intravenous immunoglobulins and systemic corticosteroids [10, 28, 29, 31, 33, 34, 36, 37, 38].

The procoagulant state caused by SARS-CoV-2 infection is another potential mechanism involved in the occurrence of cardiac lesions associated with this infection. Several studies and case reports proved that this virus has the ability to increase the risk of ischemic and thromboembolic events [5, 20]. This hypothesis is also sustained by similar effect of other coronaviruses that was proved in previous studies with a major impact on regulating the genes responsible for triggering a procoagulant state [20]. This procoagulant effect is the most expressed in patients previously diagnosed with congenital heart disease [29, 34, 37, 38]. Several studies from Italy and China highlighted that patients with preexistent cardiovascular disease, congenital or acquired have a higher risk of COVID-19-associated mortality [13, 39]. Unsurprisingly, in most of the cases this risk increases with the severity of congenital heart disease [40, 41]. Moreover, these patients usually present also with other organs affected due to their congenital malformations involving lung and renal disease, as well as cirrhosis which further increase the risk of developing severe COVID-19 forms [42]. Patients with congenital and acquired heart diseases need close monitoring since certain symptoms caused by either COVID-19 and their underlying disorders such as shortness of breath, fever and palpitation may overlap during the course of this infection indicating for example either an endocarditis or a severe form of COVID-19, and the cause of this symptoms should be carefully and early identified in order to prevent life-threatening complications [10, 43]. Disseminated intravascular coagulation was also proved to induce myocardial injury according to post-mortem biopsy reports of patients infected with coronavirus [44].

### 2.2 COVID-19 Procoagulant State in Children

This procoagulant state is no longer a myth in pediatric patients at the beginning of this pandemic era, clinicians were forced to adjust their previous knowledge for this mysterious condition and therefore several reports mentioned using antiplatelet and/or anticoagulant drugs in patients with COVID-19 and increased risk of thromboembolic events such as giant coronary aneurisms or severe disease [45]. Moreover, the International Kawasaki Disease Registry recommended the use of prophylactic dosing of anticoagulants in patients ≥12 years of age with obesity, altered mobility, preexistent thrombophilia or history of thrombosis, as well as critical presentation of COVID-19 [45]. Later, the Advanced Cardiac Therapies Improving Outcomes Network, a collaborative network dedicated to improve the outcomes of children with end-stage heart failure [46] designed an algorithm for evaluation the risk of MIS-C thrombosis including echocardiogram, electrocardiography, baseline anticoagulation laboratory tests (complete blood count, renal and liver function), tests for characterizing the inflammatory status (ferritin, von Willebrand factor antigen, erythrocyte sedimentation rate and C-reactive protein), as well as tests for characterizing the coagulation profile (Prothrombin Time/International Normalized Ratio (PT/INR), D-dimer ever 24–72 hours during admission, partial thromboplastin time, fibrinogen, lactate dehydrogenase, platelet count, thromboelastographic with platelet mapping, and thorough anamnesis for detecting history of recent or remote thrombosis, as well as family of personal history of thrombophilia) [47]. According to the previously mentioned guidelines, all these investigations should be performed at admission and at discharged, but also if the clinical status changes.

In terms of thromboprophylaxis during admission, the authors stated that patients should continue their antiplatelet or anticoagulant treatment for their preexisting conditions in the setting of COVID-19. Moreover, for those without preexisting antiplatelet or anticoagulant therapies, aspiring should be used in all patients with MIS-C except for those with platelet count <100,000/${\mathrm{mm}}^{3}$, fibrinogen <100 mg/dL, and those with active hemorrhage or high risk of bleeding [47]. The treatment should be administered at least one month or even longer if the laboratory tests remain modified. In addition to aspirin, the authors recommended full anticoagulation for patients with moderate or severe ventricular dysfunction, coronary dilation or aneurism defined by a z-score ≥10, as well as D-dimer >10x upper normal limit until the patient is able to switch to prophylactic dose as laboratory tests and echocardiogram parameters improve. Prophylactic dose of anticoagulant along with aspirin should be provided in patients with mild to moderate ventricular dysfunction, coronary dilation or aneurism with a z-score between 2.5 and 10, venous thromboembolism prophylaxis according to institutional recommendations, D-dimer between 5–10x the normal upper limit, electrocardiogram abnormalities or thromboelastographic maximal amplitude ≥80 mm. The prophylactic dose should be continued according to the underlying condition if present, and if not until the laboratory tests and echocardiography improves [47]. Antiplatelet and/or anticoagulant therapies should be continued for approximately 1 month after the diagnosis depending on the inflammatory and coagulation tests. Moreover, electrocardiogram and echocardiography should be repeated within 2 weeks after discharge if the patients was found with abnormalities during admission and serially thereafter until the normalization of modified parameters [47].

### 2.3 Risk Factors for Severe COVID-19 in Children

Taking into account the severity of cardiac lesions triggered by SARS-CoV-2 infection, the children associating these types of complications should definitely be considered with severe COVID-19 forms.

Several studies and case reports during the pandemic emphasized that young age might be considered a major risk factor for severe COVID-19 [48]. Thus, two multicenter studies performed on COVID-19 children from Europe pointed out that neonates are more likely to require intensive care unit (ICU) admission as compared to older children [49, 50]. Similarly, two case series also concluded that neonates have a higher risk of developing severe forms of COVID-19 [51, 52]. Nevertheless, the results remain controversial since other studies involving children below the age of 1 and 2 years revealed a lower prevalence of severe COVID-19 when compared to other age groups [53, 54]. Moreover, other authors also failed in identifying a significant association between neonates and the risk of ICU admission due to COVID-19 [55, 56]. A recent systematic review concluded that age does not impact the severity of COVID-19, but in terms of neonates subgroup the authors found a higher risk of severe COVID-19 as compared to other groups [48].

Prematurity, immunocompromised status and several underlying conditions such as genetic syndromes, neurological disorders, chronic lung disease, asthma, diabetes and obesity were also reported to contribute in certain cases to the development of severe COVID-19. Prematurity seems to have a great impact on the occurrence of severe forms triggered by SARS-CoV-2 infection. A study performed on COVID-19 children from the United Kingdom showed a prevalence of ICU admission of 19.2% in premature infants as compared to only 6% in full-term ones [50]. Similar findings were reported also for United States of America [57]. Immunocompromised patients were also reported to be at risk for developing severe COVID-19 [48]. In terms of immunocompromised status, children with oncological disorders seem to have the greatest chance for requiring ICU admission [58]. Moreover, those with hematopoietic stem cell transplantation were also proved to have an increased risk of severe COVID-19 [59]. Likewise, neurological disorders might also worsen the clinical course of COVID-19 in children, especially if they were previously diagnosed with seizure disorders [48]. A previous case reported by our team with a history of neurological disorder associating seizures also required ICU admission due to COVID-19 and unfortunately presented a fatal outcome [5]. Chronic lung diseases displayed the same negative impact on the severity of COVID-19 as the previously mentioned comorbidities [48]. Contrariwise, asthma seems to not influence either the clinical course or COVID-19 or the need for ICU admission in children [48, 50, 60, 61]. Children with type I diabetes mellitus were also proven to develop more severe forms of COVID-19 during the pandemics, but they did not necessarily require ICU admission [48, 50, 56, 57]. As compared to aforementioned controversial factors, obesity was found constantly to have a negative impact on the clinical course of this novel condition leading to severe COVID-19 forms in pediatric patients [48]. These findings were supported by both studies performed at the beginning of COVID-19 pandemics [50], and the recently published ones which after assessing for large samples of children (4302 COVID-19 children from USA) concluded that obesity associated an increased risk of severe forms requiring ICU admission and respiratory support, increasing at the same the mortality rate in young ages [57]. Similar findings were reported also by our team highlighting an association between obesity and the occurrence of MIS-C in a pediatric patient [4]. Contrariwise, genetic pathologies such as chromosomal abnormalities, congenital anomalies, and genetic disorders seem to have no impact on the occurrence of severe disease in COVID-19 pediatric patients, if not associated with other comorbidities [48].

### 2.4 Common Types of Cardiac Injuries

#### 2.4.1 Myocardial Impairment

A common cardiac finding triggered by SARS-CoV-2 infection, probably the most common is acute myocardial dysfunction [1, 28]. Even from the beginning of this pandemics, more than 50% of the patients diagnosed with MIS-C in United Kingdom (6/8) and Italy (5/10) were found with left ventricular dysfunction [1]. These findings were further confirmed by larger case series which indicated that up to 60% of these patients presented with decreased left ventricular ejection fraction, with a considerable risk of left ventricular dysfunction in those presenting with shock [6, 9, 28, 62]. Other case series reported in France and Switzerland diagnosed with acute heart failure due to MIS-C pointed out that although left ventricular ejection fraction was decreased in all patients, one third of them presented with severely depressed left ventricular ejection fraction, of <30%. Nevertheless, none of the patients included in the previously mentioned study died [1]. Full recovery of the left ventricular function was proved even in patients with cardiogenic or vasoplegic shock associated with a median ejection fraction of 35% requiring inotropes or vasopressors [63]. The most important predictors of left ventricular dysfunction are troponin and B-type natriuretic peptide (NT-proBNP) [1]. The level of these markers has been proven to be correlated with the need for critical care [36]. Valverde *et al*. [64] indicated that 93% of the patients with MIS-C included in their study presented elevated troponin and NT-proBNP, while only 52% of them were found with depressed left ventricular ejection fraction. A study that compared children with and without myocarditis in the setting of PIMS pointed out that those with myocarditis required significantly more frequent mechanical support as compared to those who did not develop myocardial impairment [65]. Moreover, the authors showed that children with myocarditis presented significantly higher leukocytes count in comparison to those without myocarditis. Interestingly, this study found no association between troponin levels and myocarditis. Still almost all patients, except for one, recovered the left ventricular systolic function.

According to the reports from the literature, several factors contribute to the development of myocardial impairment such as hypoxia, hypoperfusion, changes related to cytokine storm, microvascular modifications, treatment-induced cardiac toxicity and pre-existing cardiac injuries [66]. Therefore, myocarditis should be suspected in all patients presenting with acute heart failure, myocardial dysfunction, increased troponin levels and cardiogenic shock [67].

#### 2.4.2 Pericardial Impairment

Along with myocarditis, pericarditis was found in up to 25% of the patients with COVID-19 [20] and together they worsen the prognosis of this condition [68]. Moreover, the association between myocarditis, pericarditis and myocardial dysfunction characterizes COVID-19-associated pancarditis [69]. In terms of pediatric population, it was reported that approximately 20% of the cases diagnosed with MIS-C might present with mild-to-moderate pericardial effusion associated with acute cardiovascular manifestation, but severe effusion is uncommon [64]. Nevertheless, pericardial involvement in the setting of acute COVID-19 may not always associate myocardial involvement and rarely it can present as isolated tamponade requiring urgent drainage [70]. Pawar *et al. * [71] reported a case of MIS-C with myopericarditis and acute pericardial tamponade in a 13-year-old patient requiring urgent surgical pericardiostomy and drainage.

Although not as common as other cardiovascular manifestations, pericardial impairment, and even acute pericardial tamponade should also be considered in pediatric patients with acute COVID-19 or MIS-C.

#### 2.4.3 Coronary Arteries Impairment

Coronary artery dilations and aneurysm represent another severe cardiac implication of MIS-C [9, 72, 73]. Although their reported incidence varies significantly, most of the larger studies reported coronary impairment to occur in 8–24% of the patients diagnosed with PIMS [64]. It is also true that most of the patients develop small aneurysms with a z score between 2.5–5, but large or giant aneurysms might also occur with a z score above 10, as well as aneurysms that appeared only during convalescence [9, 74]. A potential explanation for these aneurysms might be related to fever or circulating inflammatory mediators resulting in a severe inflammation that disrupts the arterial wall [1]. As we already mentioned, patients with COVID-19 associated an increased risk of thrombosis, including coronary thrombosis. Coronary ischemia is also possible in patients with COVID-19 mostly due to imbalance in the oxygen supply and the associated severe inflammatory response [44]. Although rare, total occlusion of the coronary arteries resulting in myocardial infarction is also possible after COVID-19 exposure regardless of the patient’s age or his preexisting conditions. Thus, a recent case report highlighted total occlusion of the right coronary artery in a previously healthy 9-year-old boy after COVID-19 exposure, confirmed by angiography, who underwent stent implantation with favorable evolution [75]. A similar case was reported also in a 4-year-old healthy child with positive anti-SARS-CoV-2 IgG antibodies who was found with a clot occluding a giant aneurysm in the left descending artery, for which the patient received fibrinolytic agents with favorable outcome [76].

In fact, both acute COVID-19 and MIS-C may result in thrombosis and coronary artery aneurisms due to the high levels of proinflammatory cytokines which trigger a procoagulant status.

#### 2.4.4 Arrhythmias

Several types of arrhythmias were reported in patients with MIS-C or severe COVID-19 including atrial and ventricular tachycardia, as well as heart block [3, 77, 78]. It was emphasized that especially firs-degree heart block, and less common high-grade heart block commonly occur in patients with impaired left ventricular systolic function [1]. Ventricular arrhythmias along with myocarditis may be the first clinical signs of COVID-19 infection [79, 80]. Other electrocardiogram abnormalities triggered by SARS-CoV-2 infection usually consist in QT prolongation, ST segment abnormalities, and T-wave changes [28]. The few studies regarding arrhythmias in COVID-19 patients underlined a prevalence of these abnormalities in up to 16.7% of the cases [79, 81, 82]. Similar findings were reported by Hui *et al*. [83], who reported that 17 out the 41 COVID-19 patients were found with electrocardiogram anomalies. Lower rates were found by Guo *et al*. [84] indicating that ventricular tachycardia or fibrillation were present in only 5.9% of the subjects diagnosed with COVID-19 infection. Surprisingly, according to a recent study performed on children infected with SARS-CoV-2 virus, ventricular repolarization might be impaired event in those who presented no symptoms [85]. Unlike the adult population, children are less likely to develop life-threatening arrhythmias related to COVID-19 such as first-degree atrioventricular heart blocks or incomplete right bundle branch clocks, but also premature ventricular complexes or supraventricular tachycardias [86]. Another study involving pediatric patients pointed out that MIS-C is definitely associated with certain electrocardiogram abnormalities over the course of the disease including low amplitude-type changes on presentation, followed by the inversion of T-waves, especially in the precordial leads [87]. The authors highlighted also the possibility of ST-segments modification and tachyarrhythmias in the setting of MIS-C.

Several mechanisms might contribute to the abnormal cardiac rhythm involving neurohormonal and inflammatory stress, metabolic disorders, but also current therapies used in the treatment of COVID-19 such as hydroxychloroquine which affects cardiac electrophysiology [1]. Nevertheless, the precise underlying mechanisms of COVID-19 induced arrhythmias remains unclear.

#### 2.4.5 Cardiac Arrest

Cardiac arrest in COVID-19 patients was reported to overpass 10% in those admitted in the intensive care unit [88, 89]. Nevertheless, no cardiac arrests were reported in patients that did not require intensive care [88]. In addition, cardiac arrest was related with 44% in-hospital mortality [90], but it seems to be rather associated the severity of COVID-19 resulting in systemic illness which imposed the admission in the intensive care unit [88].

#### 2.4.6 Role of Imaging Tools

The initial gold standard for diagnosing COVID-19-associated myocarditis was endomyocardial biopsy [91], which should be performed early in the course of this disease involving multiple specimen biopsies for optimizing diagnosis accuracy and reducing sampling error in the setting of focal myocarditis [92, 93], but it was replace in almost all situations by magnetic resonance imaging. The histological findings of COVID-19 myocarditis resemble to those previously described in SARS and Middle Eastern respiratory syndrome due to coronavirus infection [94, 95], consisting of interstitial mononuclear inflammation infiltrates [96]. Taking into account its limitations related to the possibility of missing focal myocarditis, as well as its invasiveness, this technic is not commonly used in clinical practice.

Echocardiogram is probably one of the most frequently used imaging techniques in COVID-19 patients suspected to have myocardial impairment due to its non-invasiveness, wide availability, safety, and versatility [97]. Moreover, this imaging tool allows the assessment and quantification of global and regional systolic function, but also the monitoring of potential changes in wall thickness, cardiac chambers sizes, pericardial effusion, and most important ventricular function [92, 97, 98]. According to the European Society of Cardiology Working Group on Myocardial and Pericardial Diseases, echocardiography should be performed in all patients with a clinical suspicion of myocarditis even at presentation [92, 93]. Unfortunately, several limitations are also associated with this technique such as the lack of specificity for several echocardiographic findings, and the possibility of normal echocardiogram even in the setting of myocarditis [97, 99].

The most important non-invasive diagnosis for detecting myocardial impairment remains cardiac magnetic resonance which provides the most accurate characterization of myocardial tissue [98]. Based on Lake Loui criteria which include detection of hyperemia and early capillary leakage based on T-1 weighted early gadolinium enhancement, of regional edema on the basis of T2-weighted images, as well as detection of fibrosis and necrosis based on the late gadolinium enhancement, cardiac magnetic resonance imaging was proved to identify myocardial injuries with an accuracy of 78% [97]. The specificity and positive predictive value of this imaging tool increase in the presence of 2 out of 3 characteristics [97]. Moreover, cardiac magnetic resonance imaging is essential for differentiating myocarditis from other pathologies [97]. Cardiac magnetic resonance imaging was also used to assess the children who recovered from mild forms of COVID-19 infection and pointed out no signs of myocardial inflammation or fibrosis, as well as no evidence of functional cardiac impairment, the findings being comparable to those of healthy controls [100]. Therefore, cardiac magnetic resonance displays a great utility for assessing, quantifying and long-term monitoring cardiac damage due to COVID-19.

### 2.5 Long-Term Cardiac Outcomes

Although there is a paucity regarding longitudinal data on COVID-19-associated cardiac injuries in pediatric patients, a recent study involving 60 controls and 60 patients with MIS-C assessed cardiac outcomes at 3–4 months after the onset of this condition based on both echocardiography and cardiac magnetic resonance findings [101]. The authors concluded that functional myocardial recovery and coronary outcomes are usually good in children with multisystem inflammatory syndrome triggered by SARS-CoV-2 underlining the absence of persistent subclinical dysfunction according to the used sensitive deformation parameters.

## 3. Long COVID-19

Although several authors focused their research on characterizing pediatric Long COVID-19, the data on this particular pathology remain unclear. The Long COVID-19 syndrome in characterized by the presence of SARS-CoV-2-associated manifestations which occur or persist after the acute phase without an alternative diagnosis [102]. The most commonly reported symptoms in adults include persistent and severe fatigue, fever, cough or anorexia, but also anosmia, ageusia or neuropsychiatric manifestation [103, 104]. It is highly important to distinguish Long COVID from MIS-C. A recent Italian pediatric study pointed out a cumulative incidence of Long COVID-19 of 24.3% with an increasing pattern directly related with the severity of SARS-CoV-2 infection: 11.5% in asymptomatic children, 46.5% in non-hospitalized symptomatic children, and 58% in children that required in-hospital care [105]. It was also suggested that children aged 11–16 years are more likely to develop Long COVID-19 as compared to younger ones aged 0–5 years [106, 107]. According to the Italian intersociety consensus, the most common symptoms of Long COVID-19 in children include headache, fatigue, difficulties in focusing, sleep disturbances, myalgia, arthralgia, and abdominal pain [108]. Albeit not so common, persistent chest pain, heart palpitations, stomach pain, diarrhea, and skin lesions might also be suggestive for Long COVID-19 [108]. The prognosis of pediatric patients with Long COVID-19 is usually good and the symptoms commonly solve spontaneous, but organic symptoms require a thorough evaluation including clinical, laboratory and/or imagistic investigations. It is essential to mention that psychological support is critical in pediatric patients with Long COVID-19 [108].

## 4. Conclusions

The mechanisms of COVID-19 associated cardiac injury includes cardiomyocyte injury triggered by a severe acute inflammatory response during cytokine storm, cardiomyocytes cellular damage due to viral invasion, ischemic injury associated to severe hypoxia resulting from acute lung injury and procoagulant state, therefore an evaluation algorithm for risk of thrombosis and inflammatory status is necessary in all COVID-19 pediatric patients. Identification of risk factors for severe COVID-19 is mandatory in order to identify patients with possible cardiac injury. The most common cardiac finding triggered by SARS-CoV-2 infection is acute myocardial dysfunction. Myocarditis should be suspected in all patients presenting with acute heart failure, myocardial dysfunction, increased troponin levels and cardiogenic shock. Troponin and NT-proBNP are the most important predictors of left ventricular dysfunction and should be evaluated repeatedly. Full recovery of the left ventricular function was proved in most of the children with myocardial disfunction. Imaging techniques are important tools in COVID-19 pediatric patients suspected to have myocardial impairment. Echocardiography and cardiac magnetic resonance are able to identify contractibility disfunctions and myocardial impairment. Repeated cardiological monitoring in pediatric patients with COVID-19 is essential for identifying patients with cardiac injury.
